# Resuscitation Plus Special Issue: Cardiac arrest research

**DOI:** 10.1016/j.resplu.2023.100416

**Published:** 2023-06-21

**Authors:** Keith Couper, Siobhan Masterson, Ziad Nehme

**Affiliations:** Warwick Clinical Trials Unit, University of Warwick, Coventry, UK; Critical Care Unit, University Hospitals Birmingham NHS Foundation Trust, Birmingham, UK; Clinical Directorate, HSE National Ambulance Service, Limerick, Ireland; Centre for Research and Evaluation, Ambulance Victoria, Blackburn North, Victoria, Australia; Department of Paramedicine, Monash University, Frankston, Victoria, Australia; School of Public Health and Preventive Medicine, Monash University, Melbourne, Victoria, Australia

The cardiac arrest formula for survival describes how the relationship between medical science, education, and implementation influences patient outcomes in cardiac arrest.^1^ The best patient outcomes occur in systems that robustly implement high-quality evidence and support effective training of clinicians and members of the public. Underpinning this concept is the need to generate high-quality research to inform clinical practice. Resuscitation Plus has previously opened special editions that focus on how we train individuals in resuscitation and resuscitation systems.^2^ This special edition, by focussing on how we undertake cardiac arrest research, adds to the journal’s coverage of components of the formula for survival.

Resuscitation scientists throughout the world are committed to conducting high-quality research to improve outcomes from cardiac arrest. However, conducting high-quality cardiac arrest research is challenging. Practical and ethical challenges in conducting cardiac arrest research are driven mainly by the time-critical nature of cardiac arrest treatment, patient’s lack of capacity to make decisions about research participation, and the very high rate of mortality. Differences in the actual and the interpretation of data protection law in different jurisdictions may also be an issue. These challenges may mean that some research funders do not prioritise funding for cardiac arrest research. Data from North America shows the disconnect between the high societal impact of cardiac arrest and relative low level of investment in cardiac arrest research.^3^, ^4^

A key part of the journey towards improving survival from cardiac arrest is ensuring that cardiac arrest research uses the most robust research methods to answer clinical questions that are important to patients, clinicians, and policy makers. The Canadian James Lind Alliance cardiac arrest priority setting partnership engaged with over 400 key stakeholders to identify ten key priorities for future research.^5^ These included optimising the community response to cardiac arrest, evaluating potential interventions to improve outcomes in cardiac arrest, and identifying support needs of individuals and their family members following cardiac arrest. Addressing these research priorities will require a range of research methodologies, including randomised controlled trials, qualitative research, animal studies, and observational research.

The most efficient and effective research comes from the close collaboration between clinicians, methodologists, and patients and members of the public. For example, the randomisation process is a key challenge in designing cardiac arrest randomised controlled trials due to the need for randomisation to occur quickly; treatment delays caused by the patient enrolment process may be harmful to the patient, influence the observed treatment effect, and limit the generalisability of the population enrolled.^6^, ^7^ This has led to many trials using envelope-based systems or cluster-randomisation, despite their important methodological limitations.^8^, ^9^, ^10^, ^11^ In response to this challenge, researchers in collaboration with frontline clinicians have developed novel smartphone randomisation that might facilitate rapid individual patient randomisation in the pre-hospital cardiac arrest setting.^12^

Observational studies provide an opportunity to explore the epidemiology and outcomes of cardiac arrest.^13^, ^14^ They are particularly efficient when based on routinely collected data, such as cardiac arrest registries. Whilst observational studies may provide some insights into the efficacy of cardiac arrest interventions, their findings must be interpreted with caution due to selection bias, confounding and resuscitation time bias.^15^ Novel statistical analysis strategies, such as time-dependent propensity-scored matching, may partly address the risk of resuscitation time-bias, but cannot overcome all sources of bias.^16^

This special edition of Resuscitation Plus provides the opportunity for clinicians, researchers, patients and members of the public to share their experiences and research on optimising cardiac arrest research. We welcome submission of the following three types of papers:

(1) Narrative reviews focussed on cardiac arrest research methodology, such as a specific research methodology or concept,
(2) Primary and secondary studies on issues linked to cardiac arrest research, and
(3) Study protocols.

Reviews of cardiac arrest research methodology should focus on a specific aspect of research methodology. These narrative-style reviews (maximum word count 4,000 words) should highlight key recent research, summarise specific challenges of using that methodology for cardiac arrest researchers, and summarise key methodological messages. These reviews should be discussed with the special edition editorial team before work begins (contact email: k.couper@warwick.ac.uk) to avoid duplication across reviews.

Primary and secondary research studies should adhere to standard Resuscitation Plus formatting requirements.

We welcome the publication of cardiac arrest study protocols, such as the recently published Prehospital Optimal Shock Energy for Defibrillation randomised controlled trial and EuReCa three observational study protocols.^14^, ^17^ The journal has, in line with other journals, committed to an expedited review of protocols of externally funded studies. This expedited process comprises an editorial review that focusses only on assessing adherence to relevant reporting checklists, such as SPIRIT (Standard Protocol Items: Recommendations for Interventional Trials) checklist or PRISMA-P (Preferred reporting items for systematic review and meta-analysis protocols).^18^, ^19^ We are only able to consider study protocols that have received ethical approval, where appropriate, and where recruitment is not yet complete.

The submission portal (https://www.editorialmanager.com/resplu/) will be open for submissions until 30th November 2023. If you wish your paper to be considered for this special issue, you should ensure that the article type of “VSI: Cardiac arrest research” is selected during the submission process.

## Funding

ZN is supported by a fellowship and grant funding from the National Heart Foundation of Australia (#105690, #106763).

## CRediT authorship contribution statement

**Keith Couper:** Conceptualization, Writing – original draft, Writing – review & editing. **Siobhan Masterson:** Conceptualization, Writing – review & editing. **Ziad Nehme:** Conceptualization, Writing – review & editing.

## Declaration of Competing Interest

The authors declare the following financial interests/personal relationships which may be considered as potential competing interests: ‘KC is an associate editor of Resuscitation Plus. SM and ZN are Resuscitation Plus editorial board members’.

## References

[1] Søreide E., Morrison L., Hillman K. (2013). The formula for survival in resuscitation. Resuscitation.

[2] Nabecker S., Lockey A., Greif R. (2022). Announcement of a special issue on resuscitation education in the resuscitation plus journal. Resuscitation Plus.

[3] Coute R.A., Panchal A.R., Mader T.J., Neumar R.W. (2017). National institutes of health-funded cardiac arrest research: A 10-year trend analysis. J Am Heart Assoc.

[4] Coute R.A., Nathanson B.H., Mader T.J., McNally B., Kurz M.C. (2021). Trend analysis of disability-adjusted life years following adult out-of-hospital cardiac arrest in the United States: A study from the CARES Surveillance Group. Resuscitation.

[5] Dainty K.N., Seaton M.B., Cowan K. (2021). Partnering with survivors and families to determine research priorities for adult out-of-hospital cardiac arrest: A James Lind Alliance Priority Setting Partnership. Resuscitation Plus.

[6] Perkins G.D., Kenna C., Ji C. (2020). The influence of time to adrenaline administration in the Paramedic 2 randomised controlled trial. Intensive Care Med.

[7] Roberts I., Prieto-Merino D., Shakur H., Chalmers I., Nicholl J. (2011). Effect of consent rituals on mortality in emergency care research. The Lancet.

[8] Cheskes S., Verbeek P.R., Drennan I.R. (2022). Defibrillation strategies for refractory ventricular fibrillation. N Engl J Med.

[9] Bernard S.A., Bray J.E., Smith K. (2022). Effect of lower vs higher oxygen saturation targets on survival to hospital discharge among patients resuscitated after out-of-hospital cardiac arrest: The EXACT randomized clinical trial. JAMA.

[10] Kennedy A.D.M., Torgerson D.J., Campbell M.K., Grant A.M. (2017). Subversion of allocation concealment in a randomised controlled trial: a historical case study. Trials.

[11] Easter C., Thompson J.A., Eldridge S., Taljaard M., Hemming K. (2021). Cluster randomized trials of individual-level interventions were at high risk of bias. J Clin Epidemiol.

[12] Bloom J.E., Partovi A., Bernard S. (2023). Use of a novel smartphone-based application tool for enrolment and randomisation in pre-hospital clinical trials. Resuscitation.

[13] Nishiyama C., Kiguchi T., Okubo M. (2023). Three-year trends in out-of-hospital cardiac arrest across the world: Second report from the International Liaison Committee on Resuscitation (ILCOR). Resuscitation.

[14] Wnent J., Masterson S., Maurer H. (2022). European Registry of Cardiac Arrest – Study-THREE (EuReCa THREE) – An international, prospective, multi-centre, three-month survey of epidemiology, treatment and outcome of patients with out-of-hospital cardiac arrest in Europe – The study protocol. Resuscitation Plus.

[15] Andersen L.W., Grossestreuer A.V., Donnino M.W. (2018). “Resuscitation time bias” – A unique challenge for observational cardiac arrest research. Resuscitation.

[16] Perry E., Nehme E., Stub D., Anderson D., Nehme Z. (2023). The impact of time to amiodarone administration on survival from out-of-hospital cardiac arrest. Resuscitation Plus.

[17] Pocock H., Deakin C.D., Lall R. (2022). Protocol for a cluster randomised controlled feasibility study of Prehospital Optimal Shock Energy for Defibrillation (POSED). Resuscitation Plus.

[18] Chan A.W., Tetzlaff J.M., Altman D.G. (2013). SPIRIT 2013 statement: defining standard protocol items for clinical trials. Ann Intern Med.

[19] Shamseer L., Moher D., Clarke M. (2015). Preferred reporting items for systematic review and meta-analysis protocols (PRISMA-P) 2015: elaboration and explanation. BMJ.

